# The Role of Intracellular Trafficking of Notch Receptors in Ligand-Independent Notch Activation

**DOI:** 10.3390/biom11091369

**Published:** 2021-09-16

**Authors:** Judith Hounjet, Marc Vooijs

**Affiliations:** Department of Radiation Oncology (MAASTRO), GROW School for Oncology and Developmental Biology, Maastricht University Medical Center, P.O. 616, 6200 MD Maastricht, The Netherlands; j.hounjet@maastrichtuniversity.nl

**Keywords:** Notch signaling, intracellular trafficking, Drosophila, mammals, ligand-independent, therapeutic targeting

## Abstract

Aberrant Notch signaling has been found in a broad range of human malignancies. Consequently, small molecule inhibitors and antibodies targeting Notch signaling in human cancers have been developed and tested; however, these have failed due to limited anti-tumor efficacy because of dose-limiting toxicities in normal tissues. Therefore, there is an unmet need to discover novel regulators of malignant Notch signaling, which do not affect Notch signaling in healthy tissues. This review provides a comprehensive overview of the current knowledge on the role of intracellular trafficking in ligand-independent Notch receptor activation, the possible mechanisms involved, and possible therapeutic opportunities for inhibitors of intracellular trafficking in Notch targeting.

## 1. Introduction

Notch signaling is a highly conserved signaling pathway involved in proliferation and differentiation during embryonic development and adult homeostasis. Canonical Notch signaling involving ligand-activation, Adam metalloprotease and γ-secretase cleavage, and the role of endosomal trafficking is well described in *Drosophila*. However, the mechanisms of ligand-independent Notch signaling and the role of endosomal trafficking in mammals are still elusive. In this review, we discuss ligand-independent Notch signaling in mammals in both physiological and pathological conditions with cancer prone gain-of-function alleles. In addition, we focus on the proposed mechanisms involved in ligand-independent Notch signaling, including the role of intracellular trafficking, and the current challenges of therapeutic targeting of Notch signaling. Lastly, we discuss whether modulating intracellular transport may overcome some of these current challenges.

## 2. The Notch Signaling Pathway

**Figure 1:**
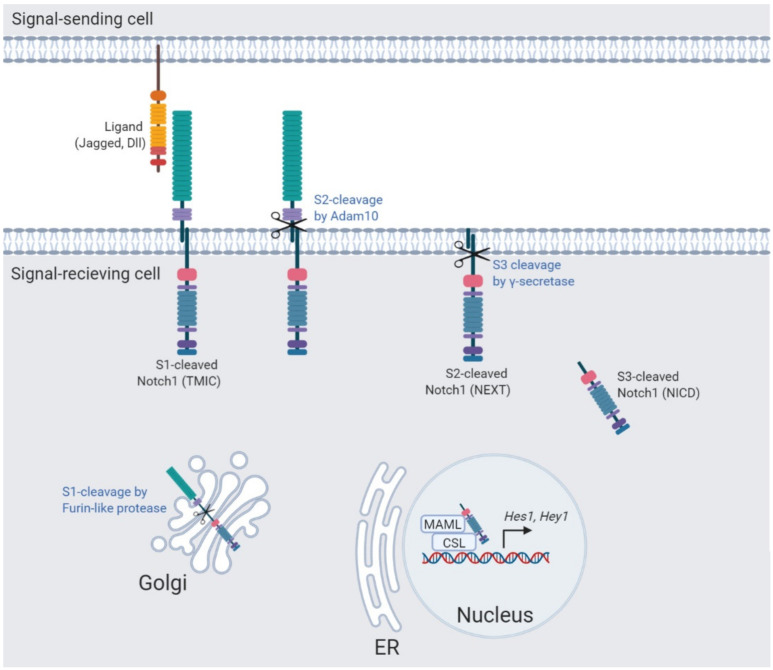
The Notch signaling pathway. Notch signaling requires ligand binding, expressed by a signal-sending cell and three sequential cleavages to release the active form of Notch, which activates Notch downstream target genes. Tmic: transmembrane intracellular fragment, Next: Notch extracellular truncation, Nicd: Notch intracellular domain.

Notch signaling is governed by three successive proteolytic cleavages (S1–S3) (Figure 1). In the Golgi apparatus, the Notch receptor is cleaved at the S1-site by a furin-like protease creating a non-covalently associated Notch heterodimer composed of the Notch extracellular domain, the transmembrane, and intracellular domain (Tmic), which is transported towards the plasma membrane, where it awaits ligand binding [1]. In the absence of ligand, the extracellular S2-cleavage site is masked by the negative regulatory region (NRR) composed of the heterodimerization (HD) domain and Lin12-Notch repeats (LNR), leading to the auto-inhibition of the Notch receptor [2]. Upon ligand binding, the NRR unfolds and reveals the S2-site, which is subsequently cleaved by the metalloprotease Adam10 resulting in a membrane-tethered truncated Notch receptor (Next) [3,4]. To complete the Notch signal transduction route, the Notch receptor is cleaved at Val1744 (S3) in the Notch transmembrane domain by the intra-membrane γ-secretase complex, releasing the Notch-intracellular domain (Nicd). The Nicd is transported towards the nucleus where it associates with a transcriptional complex containing the RBP-jκ/CSL DNA binding protein to activate its downstream targets [1]. For many years this was thought to be the predominant route of Notch activation, however, evidence of intracellular trafficking regulating Notch receptor activation at several steps of the signal transduction route is increasing.

## 3. Intracellular Trafficking of Transmembrane Proteins

**Figure 2:**
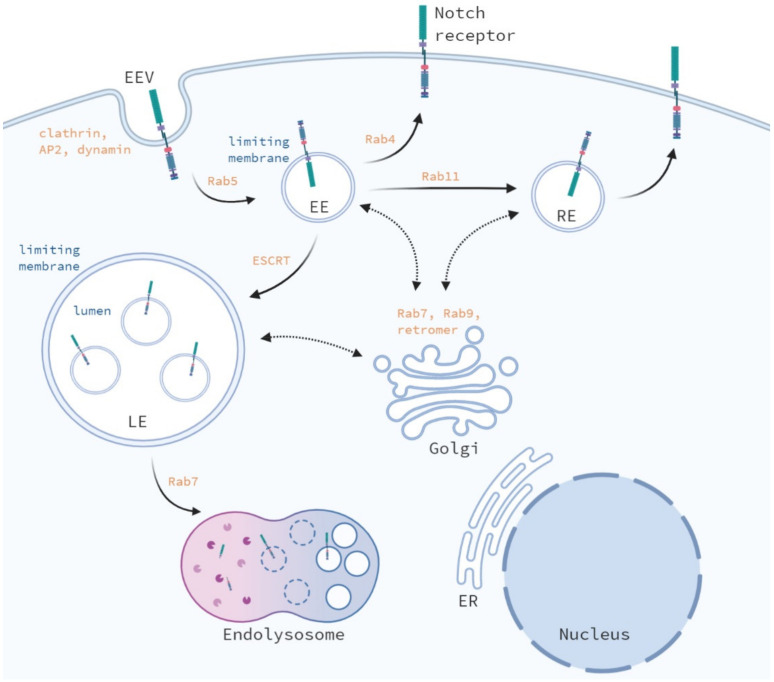
The role of endocytosis in the Notch receptor signaling. The Notch receptor is continuously internalized. While the majority of internalized Notch receptors are directly transported back to the plasma membrane, the minority of internalized Notch receptors are further transported towards the endocytic compartments and either recycled to the plasma membrane via recycling endosomes or degraded in the lysosomes. EEV: early endocytic vesicle, EE: early endosomes, RE: recycling endosomes, LE: late endosomes.

One of the first demonstrations of receptor-mediated endocytosis was the degradation of the low density lipoprotein (LDL) [5]. LDL was shown to be degraded by a mechanism of clustering at the cell surface, binding to surface protein clathrin, and subsequently pinching an internalization of vesicles targeted for degradation. Clathrin-mediated endocytosis starts with the adaptor protein 2 (AP2) complex, which recruits clathrin to the plasma membrane initiating the formation of a clathrin-coated pit. The AP2 complex and additional clathrin-associated sorting proteins act as adaptor proteins and regulate cargo recognition and sorting. Next, the clathrin-coated pit grows and matures into a clathrin-coated vesicle, also called early endocytic vesicle (EEV) (Figure 2). Subsequently, the EEV is pinched of by the GTPase dynamin, which releases the clathrin-coated vesicle into the cytosol. Next, the released clathrin-coated vesicle is uncoated by the ATPase heat shock cognate 70 (HSC70) and auxilin and the EEV can fuse with other EEVs or early endosomes (EEs) [6]. 

Clathrin-independent mechanisms of endocytosis have also been reported. One of these is caveolae-mediated endocytosis. Caveolae are invaginations in the plasma-membrane enriched in cholesterol and sphingolipids and also called “lipid rafts”. Like clathrin-mediated endocytosis, caveolae-coated vesicles are also pinched off by dynamin, however, regulators of lipid raft formation and budding are still elusive. Other clathrin-independent mechanisms of endocytosis have been reported, involving: endophilin, RhoA, Rac1, Cdc42, Arf6, and flotillins [7]. To date, it remains unclear whether these endocytic pathways are mechanistically distinct and future research has to clarify to which extent they contribute to the endocytic capacity of cells. 

Subsequent intracellular trafficking of vesicles is regulated by Rab proteins, which are a large family of small GTPases. Early endosomes are weakly acidic (pH 6.8–5.9) [8] and function as the main sorting station, in which cargo is retained, sorted, or accumulated by the regulation of Rab5, before fusing with late endosomes [9]. During endosome maturation cargo sorting and the formation of intraluminal vesicles is regulated by ESCRT complexes. Moreover, endosomal and lysosomal proteins expressed by intracellular vesicles are continuously exchanged between endosomes and the trans-Golgi system, which is regulated by Rab7, Rab9, and the retromer complex during endosome maturation [10,11]. Furthermore, Rab5 recruits Rab7 leading to the formation of Rab5/Rab7 expressing endosomes from which Rab5 is subsequently removed during the “Rab switch”. This Rab switch enables the proper association and dissociation of adaptor proteins regulating the endocytic pathway, in which Rab7 is the major regulator in late endosomes and lysosomes. Mature late endosomes (LEs) exist of a limiting membrane expressing LAMP1 and intraluminal vesicles and acid hydrolases in the lumen. Late endosomes travel from a cytosolic region to a perinuclear region, where they fuse with other late endosomes, lysosomes, or endo-lysosomes for cargo degradation [12,13]. 

Alternatively, early endosomes can also recycle back to the plasma membrane via Rab4, or can first fuse with a recycling endosome and subsequently fuse with the plasma membrane, a process requiring Rab11 [14]. Recycling of unbound Notch ligands and endocytosis of Notch ligands bound to the extracellular domain of the Notch receptor play a critical role in Notch receptor processing and activation [15,16,17]. Ligand-endocytosis may play a role in both endocytosis-dependent and independent Notch signaling if ligand and receptor are transported on vesicles in close proximity. In this review we focus on the role of endosomal trafficking of the Notch receptor instead of its ligands. 

## 4. Endosomal Trafficking of the Notch Receptor; A Comparison between *Drosophila* and Mammals

In *Drosophila*, several endocytic regulators tightly control the intracellular trafficking of the Notch receptor and interruption of the different steps of intracellular trafficking have different effects on both ligand-dependent and independent Notch signaling activation. Although Notch signaling is a highly conserved pathway, there are significant differences between Notch signaling in *Drosophila* and mammals. First, the *Drosophila* genome encodes only one Notch receptor, while mammals express four Notch receptors. In addition, Notch signaling in *Drosophila*, does not require furin-cleavage for cell surface expression and ligand activated proteolysis [18]. In mammals, loss of furin-cleavage of Notch1 results in decreased surface expression and activity, while loss of furin-cleavage of Notch2 does not affect surface expression or activity of Notch2 [19]. Despite these differences between *Drosophila* and mammals, is the intracellular trafficking and regulation of the Notch receptor conserved between these species?

While intracellular trafficking of the Notch receptor is well studied in *Drosophila*, its role in Notch signaling in mammals is less clear. In mammals, endocytic regulation is more complex because most regulators known in *Drosophila* have multiple orthologs in mammals. Moreover, many of these regulators are essential for maintaining tissue homeostasis and therefore loss-of-function often leads to early lethality. An overview of the key regulators involved in endosomal and lysosomal trafficking and the effect on Notch signaling upon their loss-of-function is summarized (Table 1).

### 4.1. Internalization of the Notch Receptor

Although Notch ligands and receptors are predominantly transported by clathrin-mediated endocytosis in mammals, the Notch receptor can also be internalized by clathrin-independent endocytosis [20,21]. In mammals, the majority of γ-secretase complexes are in lipid rafts, preventing the cleavage of Notch1 [20]. However, Notch1 receptors can also be found on lipid rafts expressing caveolin-1 [21], although Notch cleavage was not active in lipid rafts [20].

Loss of Pofut1, which mediates the O-fucosylation of the Notch receptor, results in an accumulation of Notch receptors in the ER, caveolin-expressing vesicles, and intracellular vesicles yet to be identified. Inhibition of caveolin-1 results in increased γ-secretase activity, Notch processing, and target gene expression, due to increased co-localization of γ-secretase on clathrin-coated vesicles. In contrast, ectopic expression of caveolin-1 increased the co-localization of γ-secretase (Nicastrin and Presenilin 1) with calveolin-1-coated vesicles [22]. Interestingly, the catalytic domains of the γ-secretase complex; Psen1 and Psen2, also showed a distinct intracellular localization [23].

Although the majority of PSEN1-containing complexes were expressed at the cell surface, PSEN2-expressing complexes were located to intracellular vesicles, which transport route may also be differentially regulated by using either calveolin-1 or clathrin-expressing vesicles for transport. Together these data imply that Notch activation is tightly regulated by the spatial separation of Notch receptors and the γ-secretase complex by caveolin-mediated endocytosis in mammals.

In general, loss of function of regulators of the early endosomal formation and trafficking, including *Dynamin*, *Clathrin*, *Rab5* or *Avl*, lead to an accumulation of the Notch receptor at the plasma membrane and a loss of activation in *Drosophila* [24,25,26]. Mammals express three orthologs of Dynamin; Dynamin 1, 2 and 3. Loss of Dynamin1 results in decreased Notch1ΔE (truncated membrane-tethered Notch1 without S2-cleavage site) internalization without affecting Nicd-Val1744 cleavage and translocation to the nucleus in Hela cells [27]. However, Notch target gene activation was not assessed and Dynamin1 is predominantly expressed in the brain and therefore studying its role in cervical cancer cells may be less relevant. In contrast, loss of Dynamin2, which is ubiquitously expressed, prevents S3-cleavage of Notch1ΔE by γ-secretase thereby blocking Notch1 signaling [28]. Inhibition of clathrin heavy chain also resulted in decreased Notch1ΔE internalization without affecting Val1744 levels or its nuclear localization [27], however, expression of downstream targets of Notch was not assessed. These data suggest that the endocytosis-dependent Notch pathway is perturbed upon loss of clathrin in mammals, however, activation of endocytosis-independent Notch signaling may mask these affects. Dynamin triple knockout fibroblasts show severe defects in clathrin-mediated endocytosis, however, no analysis on Notch signaling has been reported yet [29]. In mammals, four different clathrin orthologs are known, including: clathrin light chain a and b and clathrin heavy chain 1 and 2. Therefore, loss of the different orthologs could have different effects on Notch signaling and may compensate partially for loss of these orthologs in a context dependent manner.

An additional key regulator of Notch receptor internalization in both *Drosophila* and mammals is Crumbs. Crumbs is a transmembrane protein, which regulates epithelial polarity and reduces Notch activity by preventing the Notch receptor internalization [30], resulting in reduced γ-secretase cleavage of Notch in *Drosophila* [31]. Upon loss of Crumbs, Notch internalization increases, leading to ligand-independent activation. In mammals, there are three orthologs of Crumbs, which all decrease Notch activation upon ectopic expression [32]. Mechanistically, Crumbs orthologs encode EGF-like repeats, which bind to the ligand-binding domain of Notch to compete with Notch ligands preventing its internalization and subsequent activation. Crumbs inhibition on Notch works in cis (when expressed in the same cell) but not in trans.

### 4.2. Recycling of the Notch Receptor

The majority of internalized Notch receptors are directly transported back to the plasma membrane mediated by Rab4 (Figure 2). The cargo is sorted by the retromer complex, composed of a cargo-sorting sub complex (Vps35/Vps26/Vps29) and a sorting nexin sub complex [33]. A fraction of the internalized Notch receptors in early endosomal vesicles fuse with early endosomes via Rab5 or are indirectly transported back to the plasma membrane via Rab11-positive recycling endosomes. The receptor-mediated protein 8 (Rme-8), or DNAJC13 (human ortholog), regulates intracellular trafficking by cargo sorting, transport from the endosomes towards the Golgi network, and receptor recycling in both *Drosophila* and mammals [34,35,36]. In *Drosophila*, depletion of Rme-8 leads to an accumulation of both full length and S3-cleaved Notch receptors in enlarged Rab4+ endosomes. This disturbance in Notch receptor trafficking results in a decreased number of Notch receptors at the plasma membrane and reduced Notch activity [37]. The effect on Notch receptor trafficking upon loss of DNAJC13 in mammalian cells remains unstudied.

Numb is an endosomal adaptor protein, which regulates the balance between internalized Notch in recycling endosomes and late endosomes. Upon loss of Numb signaling, Notch entry into late endosomes decreases, which leads to an accumulation of Notch at the plasma membrane, due to increased recycling, and decreased degradation, leading to increased Notch activation [38,39]. Numb regulates the distribution of α-adaptin, which controls Notch receptor-mediated endocytosis and lysosomal degradation [40]. In *Drosophila*, the lethal giant larvae (Lgl) gene encodes a myosin II-binding protein, which acts a tumor suppressor by regulating proliferation and tissue integrity. Lgl acts upstream of Numb and regulates the localization and activation of Numb in daughter cells during cell division, regulating cell fate by local inhibition of Notch signaling [41]. In mammals, Numb also negatively regulates Notch activation by promoting the ubiquitination of Notch1 receptors at the plasma membrane and their subsequent degradation [42]. Loss of Numb has been reported in human malignancies, including breast and lung cancer, leading to increased Notch signaling [43,44]. In mammals, asymmetric localization of Numb is lost upon loss of Lgl expression, which results in increased Notch cleavage and target gene activation [45].

Recycling of Notch2 at the cell surface is regulated by the Commd9 and retromer complexes, in which Commd9 prevents lysosomal degradation of Notch2 receptors [46]. Upon loss of Commd9, Notch2 receptors accumulate in Rab5+ endosomes, cell surface expression is decreased, and expression in recycling Rab11+ endosomes is reduced. Consequently, Notch target gene activation is inhibited by enhanced transport of Notch2 to the lysosomes and subsequent degradation.

### 4.3. Early Endosomal Trafficking

Once incorporated into early endosomes the Notch receptor is localized on the limiting membrane, in which the Notch intracellular domain (Nicd) protrudes towards the cytoplasm [47,48,49,50] (Figure 2). During the maturation of early endosomes into late endosomes, membrane-bound proteins can selectively be packaged into intraluminal vesicles by ESCRT proteins leading to the formation of multivesicular bodies (MVBs). This selection process is regulated by ubiquitination of Notch by the E3 ligases *Deltex* (*Dx*), *Suppressor of Deltex* (*Su*(*dx*)) and *Kurtz* (*Krz*) in *Drosophila* [48,51,52]. Mono-ubiquitination of the Nicd by *Dx* blocks transport to MVBs, resulting in stabilization of Notch in maturing endosomes, which leads to Notch activation. However, Dx-mediated Notch signaling is tissue specific and whether its effects results in inhibition or activation of Notch signaling may depend on its interacting partners [53]. First, Dx activates Notch signaling independent of ligand binding and transports the Notch receptor from the cell surface towards the late endosomes, which requires Rab5. Loss of Dx results in a decreased number of Notch receptors in endosomes [54]. Deltex, can also form a protein complex consisting of Notch, Deltex, and Kurtz (β-arrestin), by direct binding to the ankyrin repeats present in Nicd, which targets Notch for degradation [55].

In mammals, there are five *Dx* orthologs, from which Dtx1, Dtx2 and Dtx4 bind Nicd1 [56,57]. While Dx is a positive regulator of Notch signaling in *Drosophila*, Dtx1 and Dtx2 are negative regulators of ligand-dependent Notch signaling during T-cell development in mammals. All three Deltex homologues (Dtx1, Dtx2, and Dtx4) are expressed by thymocytes and are able to block Notch signaling in mammals although how is not clear [58]. If Deltex can also activate mammalian Notch signaling remains unclear. Mammalian Dtx1 was shown to directly induce the transcription of Notch target genes by direct binding to the transcriptional co-activator p300 in the nucleus [59]. Loss of function studies in mammals showed that loss of Dtx1 does not affect T-cell development, which may result from a redundancy between the three Deltex homologs. However, loss of both Dtx1 and Dtx2 in mice increased Notch signaling, however, did not affect T-cell development, which suggests that Dtx1 and Dtx2 are negative regulators of Notch signaling in T-cells, but are not essential for Notch signaling during T-cell development [58]. Loss of both Dtx1 and Dtx2 resulted in an increase in Dtx4 expression, which may have masked the effects on T-cell development, however, additional loss of Dtx4 showed also no significant changes in T-cell development. Dtx4 was shown to be a potent enhancer of ligand-dependent Notch1 signaling by ubiquitination of Notch1 upstream of Adam10 [57]. Other protein interacting partners may induce differences in Notch activation in *Drosophila* and mammals.

On the other hand, poly-ubiquitination of Nicd by *Su* (*dx*) or *Krz* is recognized by ESCRT proteins, resulting in the transport of Notch into multivesicular bodies, in which the Nicd of the Notch receptor no longer protrudes into the cytosol (Figure 2). MVBs can either fuse with lysosomes mediated by Rab7, leading to Notch degradation or fuse with the plasma membrane leading to the secretion of extracellular vesicles [60]. While loss of *Dx* results in loss of Notch internalization and signaling [61], inhibition of *Su* (*dx*), an E3 ligase regulating *Dx* degradation, leads to increased Notch signaling in *Drosophila* [62,63]. Similar effects on Notch signaling were observed upon loss of DNedd4, a second member of the *Drosophila* Nedd4 family [52]. In *Drosophila*, Nedd4 binds and ubiquitinates Nicd, which results in Notch trafficking into early endosomes and targets Notch and Deltex for degradation [64]. Loss Nedd4 reduces Notch receptor internalization and activates ligand-independent Notch signaling. The mammalian ortholog of *Drosophila Su* (*dx*), AIP4/Itch, also poly-ubiquitinates the Nicd in the absence of ligand by indirect binding, leading to its lysosomal degradation [65]. This indirect binding was not enhanced by Dtx or Numb overexpression, although Numb and Itch interact with each other to promote the ubiquitination of Notch1 localized at the plasma membrane [42]. Therefore, other factors or post-translational modifications might facilitate the interaction between Notch and Itch. AIP4/Itch also partially co-localizes with Dx in endocytic vesicles and poly-ubiquitination of Dx by Itch targets it for lysosomal degradation [66]. However, loss of Itch showed no ectopic activation of the Notch receptor [65].

### 4.4. Secretion via Exosomes?

Recently, Notch ligands have been found in the membranes of exosomes secreted by endothelial cells [67], mouse embryonic stem cells [68], and mesenchymal cells [69]. In addition, Notch receptors (Notch1 and Notch2), Adam10, and Itch have been reported to be secreted in arrestin domain-containing protein 1 (ARRDC1)–expressing extracellular vesicles [70]. In addition, cleaved Notch2 receptors (S2- and S3-cleaved) but not full length Notch2 have been detected in exosomes. Knockdown of Itch or Adam10 resulted in loss of cleaved Notch2 in exosomes. Exosomal Notch2 increased the expression of Notch target genes in recipient cells, which was diminished upon γ-secretase inhibition of the recipient cells. These data imply that long range Notch signaling can be transferred via Notch ligands and Notch receptors in exosomes.

### 4.5. Late Endosomal and Lysosomal Trafficking

The inhibition of the maturation of early endosomes into MVBs, for example by loss of *ESCRT* or *lethal giant discs* (*lgd*), results in a retention of Notch in endosomes, leading to ligand-independent activation of Notch in *Drosophila* [25,71]. *Lethal giant discs* (*lgd*) is a tumor suppressor gene in *Drosophila* and a member of an uncharacterized protein family. In *Drosophila*, *lgd* mutant cells have enlarged mature endosomes and activation of endocytosis-dependent Notch signaling [50]. This ectopic activation of Notch signaling requires both γ-secretase and V-ATPase activity and localizes to the lysosomes. Schneider et al. suggest that the endocytosis-dependent activation of Notch also requires the fusion of mature endosomes with lysosomes since Rab7 depletion reduces the ectopic Notch activation in *Lgd* mutant cells. In contrast, Rab7 depletion upon loss of ESCRT protein expression has no effect on endocytosis-dependent Notch signaling, while loss of ESCRT proteins activates endocytosis-dependent Notch signaling, indicating that *ESCRT* and *Lgd* mutants activate endocytosis-dependent Notch signaling by different mechanisms.

Mammals have two *Lgd* orthologs: Cc2d1a and Cc2d1b. Loss of Cc2d1a, but not Cc2d1b, in the mouse intestine leads to an increased size of endosomes/lysosomes [72]. However, no effect on Notch signaling was observed on secretory differentiation (i.e., goblet cell number) or target gene expression. Several loss-of-function studies on ESCRT proteins were performed. While loss of CHMP5 (required for ESCRT-III activity) and Vps25 (component of ESCRT-II) did not activate Notch signaling, loss of Tsg101 (component of ESCRT-I) results in ligand-independent activation of Notch in vitro [73]. However, ligand-independent activation of Notch upon loss of Tsg-101 in vivo could not be studied due to early embryonic lethality. Moreover, loss of basal body proteins (BBS1, BBS3, and BBS4), which all interact with Tsg101, leads to increased endocytosis-dependent Notch signaling due to decreased Notch cell surface expression and increased localization in the late endosomes and reduced lysosomal degradation [73].

Ligand-independent activation of Notch signaling after disruption of endosomal and lysosomal trafficking occurs in lysosomes, were Notch receptors are localized to the limiting membrane of the lysosomes [49]. Here, γ-secretase complexes are also present and cause cleavage and release of Nicd into the cytoplasm, which results in target gene activation. In mammals a highly conserved dileucine sorting motif, localized between the ankyrin repeats and TAD-domain of the Nicd, was identified which targets the Notch receptor to the limiting membrane of the lysosomes and prevents Notch receptor degradation in the lysosomal lumen [74].

However, interference with the acidification of endosomes and lysosomes by the loss of *V-ATPase* or *Big brain* expression, results in an accumulation of Notch in enlarged late endosomes and lysosomes and a decrease in both endogenous and ectopic Notch signaling [75,76,77,78]. Although Lgl negatively regulates Notch activation by regulating the localization and activity of Numb, Lgl also inhibits endocytosis-dependent activation of Notch signaling by reducing endosomal vesicle acidification. Mechanistically, Lgl interacts with Vap33, which binds and inhibits V-ATPase activity, reducing endocytosis-dependent Notch activity [79]. In line, loss of *Lgl* expression showed increased Notch activity, due to decreased endosomal pH and increased γ-secretase activity [80,81,82]. In mammals, similar effects on Notch activation were observed upon intravesicular pH modulation. In immortalized human non-tumorigenic breast epithelial cells (MCF10-A) inhibition of V-ATPase activity by Bafilomycin A1 decreased Notch signaling [76], but did not affect cell growth. However, growth of human breast cancer (HCC2218 and HCC1599) and T-ALL cells, expressing a ligand-independent Notch1 (membrane-tethered, active Notch), is inhibited by Bafilomycin A1 or γ-secretase inhibitors [76,83]. In contrast, breast cancer cells expressing a constitutively active truncated Notch2 protein, which does not include the S3-cleavage site (HCC1187), do not depend on endocytosis and are unaffected by V-ATPase inhibition.

### 4.6. Nuclear Translocation of Nicd and Notch Activation

Although nuclear import of the Nicd is essential for Notch activation, the exact mechanism remains unknown. In *Drosophila*, nuclear import of Nicd is regulated via the importin-α3/β transport system [84]. In this transport system, importin-α3 binds directly to the nuclear localization signal (NLS) of Nicd and to importin-β, which interacts with the nuclear pore complex (NPC). In mammals, importin-α3 also binds directly to one of the nuclear localization signals in the Nicd for nuclear import of Nicd. In mammals importin-α4 and importin-α7 are also involved in the nuclear translocation of Nicd. Silencing of importin-α3, α4, and α7 reduced nuclear import of Nicd in vivo [85]. In addition, phosphorylation of Nicd1 by PKCζ enhances Notch signaling by relocating Notch1 from the endosomes to the nucleus [86]. Together, these data show that the nuclear translocation of the Nicd is regulated by the importin-α3/β signaling in both *Drosophila* and mammals. In mammals additional importins (α4 and α7) are involved in the nuclear import of the Nicd, preceding Notch signaling activation.

Altogether, these data show that also in mammals both ligand-dependent and independent Notch signaling requires endocytic trafficking and interfering with the different steps of intracellular trafficking has distinct effects on Notch signaling. Does ligand-independent signaling rely more on intracellular trafficking compared to physiological Notch signaling? Moreover, could we interfere with ligand-independent by targeting endosomal/lysosomal trafficking without affecting ligand-dependent signaling?

## 5. Ligand-Independent Notch Signaling in Cancer

Gain-of-function mutations in *Notch1* are hallmark driver mutations in T-cell acute lymphoblastic leukemia (T-ALL) leading to ligand-independent Notch1 signaling [87]. Due to the high frequency of Notch1 mutations in T-ALL, γ-secretase inhibitors (GSI) have been investigated to target the Notch1 pathway in patients. Unfortunately, GSI have shown limited anti-leukemic activity in patients due to dose-limiting toxicities in normal tissues, including severe goblet cell metaplasia in the intestine and other side-effects as observed in animal models [88,89]. Notch signaling has also been linked to other types of leukemia in which *Notch1* and *Notch2* gain-of-function mutations are also reported [90].

Activating *Notch* mutations have also been reported in breast cancer. Different rearrangements in *Notch1* and *Notch2* were found in a subset of breast cancer patients and cell lines, mostly triple negative breast cancers [91]. These rearrangements result in membrane-tethered truncated Notch1 proteins, which no longer have a S2-cleavage site and therefore are only regulated by γ-secretase activity. The rearrangement in *Notch2* leads to a truncated, cytoplasmic Nicd, which no longer requires γ-secretase cleavage to become transcriptionally active. Moreover, high expression of Notch1 was associated with a poor patient prognosis, while high levels of Notch2 are correlated with a better patient outcome [92,93]. Besides mutations in *Notch1* and *Notch2*, approximately 50% of all human breast cancers show loss of Numb signaling, due to ubiquitination and degradation of Numb, which act as a tumor suppressor [44]. In addition to direct regulation of Notch activity and degradation, Numb also regulates Mdm2, which is a E3 ligase and master regulator of TP53 stability. Therefore, loss of Numb in breast cancer also results in loss of TP53 [94]. In addition, loss of Numb expression or reduced expression of Numb in primary breast tumors correlates with poor disease free survival and a higher risk of developing metastases [95] and is mainly found in triple negative breast cancers.

Alterations in the Notch pathway have also been found in lung cancer. In approximately 30% of all human non-small lung cancers (NSCLC) *Numb* expression is also lost leading to the activation of Notch1 signaling. In addition to loss of Numb, *Notch1* gain-of-function mutations are found in approximately 10% of all NSCLC patients, which localize to the heterodimerization, transactivation, and PEST domain of *Notch1* [43]. In contrast, another study showed loss-of-function mutations in all Notch receptors, although mutations in *Notch1* were most frequent (25% of human small-cell lung cancer (SCLC)) [96]. The loss-of-function mutations in *Notch1* located mainly to the extracellular domain with no specific domain preference and show that Notch signaling acts as a tumor suppressor in SCLC.

Altogether, these data show that Notch signaling is deregulated in a broad range of human malignancies either by mutations in *Notch* (only in a limited number of cancers) or by loss of negative regulators leading to (mainly) ligand-independent, constitutively active Notch signaling. Although aberrant Notch signaling has also been reported in other pathologies, including cardiovascular and liver diseases, a role for ligand-independent Notch signaling activation has not been described as far as we are aware. However, does ligand-independent Notch signaling only occur in cancers due to genomic mutations and rearrangements or is there also a physiological role for ligand-independent Notch signaling without the need for activating mutations?

## 6. Physiological Ligand-Independent Signaling in Mammals?

Physiological ligand-independent signaling has been reported in crystal cells in *Drosophila*. Crystal cells are platelet-like cells that are part of the fly immune system. These cells require Notch activity for their survival. This Notch signaling acts independently of ligand but requires endosomal trafficking of Notch [97]. Mechanistically, crystal cells express high levels of nitric oxide synthase 1 (NOS1) under normoxic conditions, which increases nitric oxide levels. Nitric oxide stabilizes HIF-α under normal oxygen levels, which leads to the accumulation of full-length Notch receptors in early endocytic vesicles, without activating hypoxia response targets. HIF-α mediated Notch activation was shown to act ligand-independently and required γ-secretase activity. A plausible explanation for this endogenous need of ligand-independent Notch activation may be the lack of ligand present. If there is no guaranteed source of Notch ligand while active Notch signaling is required for survival, as is the case for circulating blood cells, an alternative activation of Notch signaling, independent of ligand is essential. However, is endogenous ligand-independent Notch signaling only restricted to *Drosophila* or is the phenomena also present in mammalian cells?

**Figure 3:**
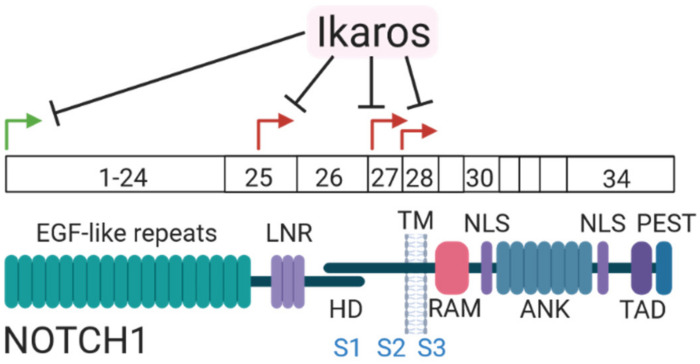
Endogenous ligand-independent Notch signaling in mammals. Ikaros regulates the physiological expression of Notch1 of both canonical and alternative transcripts. Adjusted from Gómez-del Arco, et al [98].

In mammals, ligand-independent Notch1 signaling has been reported during T-cell development in mice [98]. This ligand-independent Notch1 signaling is the result of an alternative use of the *Notch1* promoter leading to the expression of alternative transcripts downstream of the canonical promoter (Figure 3). These transcripts encode a truncated, membrane-tethered Notch1 variant. Ikaros, which is required for proper lymphoid differentiation of early hematopoietic progenitors, directly regulates the epigenetic state of the canonical and alternative promoters of *Notch1*, where it acts as a negative regulator of Notch1 signaling. Loss of Ikaros during T-cell development leads to aberrant Notch1 activation and rapid development of T-ALL. Together these data prove that ligand-independent Notch signaling does not only drive carcinogenesis, but can also occur during physiological signaling in mammals. Whether ligand-independent Notch signaling is used during additional steps of mammalian development or homeostasis during adult life remains elusive.

## 7. Mechanisms of Ligand-Independent Notch Signaling

First, what are the similarities and differences of ligand-dependent and independent Notch signaling? Both ligand-dependent and ligand-independent signaling require S3-cleavage by γ-secretase to become active [99,100,101,102]. Although, deletions in the *Notch2* receptor gene reported in breast cancer, leading to the expression of a truncated Notch receptor (Nicd-like), no longer require S3-cleavage. A second requirement for the activation of both ligand-dependent and ligand-independent Notch signaling are O-fucosyltransferase-1 Pofut1 [103] and O-glucosyltransferase Rumi [104], which are two proteins involved in the glycosylation of the EGF-like repeats of the Notch receptor that enable and regulate ligand binding. Loss of Pofut1 leads to a loss of all ligand-dependent and ligand-independent Notch1 (T-ALL mutants) signaling [103]. Loss of Rumi suppressed ligand-independent Notch signaling in *Drosophila* [105] and Rumi expression is also found to be highly up-regulated in NSCLC patients and is a predictive marker of poor patient prognosis and survival [104].

**Figure 4:**
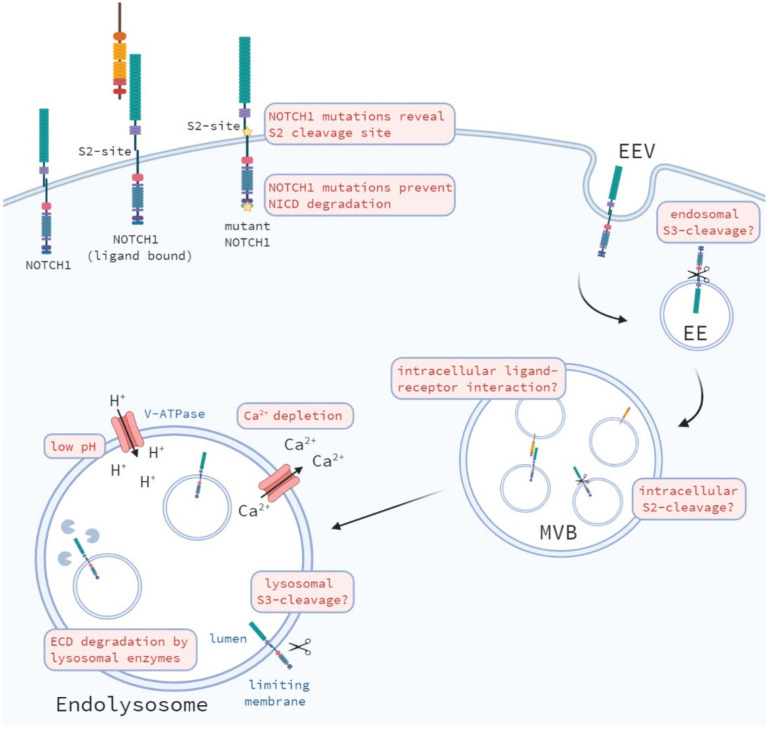
Possible mechanisms of ligand-independent Notch signaling. Ligand-independent Notch signaling can be elicited by mutations in the Notch receptor. Other mechanisms of intracellular ligand-independent Notch activation have been proposed, including low pH, Ca^2+^ depletion, lysosomal activation by degradation of the ECD of the Notch receptor, and intracellular S2- and S3-cleavage of the Notch receptor.

Ligand binding leads to conformational changes in the NRR of Notch, which result in the unmasking of the S2-cleavage site for cleavage by Adam proteases. Moreover, endocytosis of the Notch ligand bound to the Notch receptor makes sure the extracellular domain (ECD) of the Notch receptor is internalized and removed after S2 cleavage preparing the receptor for further processing by γ-secretase [106,107]. Accumulating evidence shows that S2-cleavage and removal of the NRR is essential for Notch activation [3,4,108]. Therefore, without ligand binding an alternative mechanism of shedding of the ECD of the Notch receptor is required and/or conformational changes to make the S2-cleavage site accessible for cleavage. In addition to ligand binding, ligand-dependent Notch signaling requires Adam10 activity for endogenous Notch activation, which is not essential for a ligand-independent signal [3,109,110]. Notably, Notch cleavage at Val1711 upon ligand-stimulation was reduced, however, not absent in Adam10 KO cells [3,109] and was lost upon metalloprotease inhibition [109]. Moreover, residual ligand-independent Notch activity, induced by T-ALL mutations, is observed upon loss of Adam10 and Adam17, which may be processed by other proteases yet to be identified [3,109,110]. In addition, ligand-independent Notch1 S2 cleavage was detected, although Val1711 levels were absent, indicating that additional proteases may perform S2-cleavage at a different cleavage site. However, how does Notch become active without ligand binding and Adam10 cleavage? As ligand-independent Notch signaling requires no ligand binding, the effect of ligand binding; unfolding and removal of the ECD of Notch, has to be managed by a different mechanism. Several models explain this ligand-independent activation of Notch signaling (Figure 4).

### 7.1. Mutations in the Notch Receptor

Mutations in the *Notch* receptor localizing to the heterodimerization domain, which are found almost exclusively in T-cell leukemia’s and mostly affect Notch1, are one of the mechanisms of ligand-independent Notch signaling [87,91]. Due to the mutations in the heterodimerization (HD) domain, the Notch receptor undergoes a conformational change. Mutations in the HD domain can be divided into two classes: (1) mutations which destabilize Notch1 heterodimers or (2) mutations which do not affect heterodimer stability, but enhance the access of metalloproteases to the S2-site which both increase Notch1 signaling activity ligand-independently [111]. In addition, mutations in the PEST domain of the Notch receptor, which are also found in T-ALL and often in combination with a HD mutation, prevent rapid degradation of the active Nicd, leading to a constitutively cleaved and activated Notch receptor which degradation is partially inhibited. However, human Notch2 activity is not affected by NRR disruption and ligand-independent activation by Adam17, which may explain the low frequency of Notch2 mutations [112]. Moreover, auto-inhibition by the NRR of the Notch3 receptor is less tightly associated compared to the NRR of Notch1, leading to increased basal ligand-independent Notch3 activity [113]. The mechanism of Notch4 activation has not been resolved, but Notch1-3 receptors all undergo the paradigm of sequential cleavage by a metalloprotease and subsequent cleavage by γ-secretase to produce Nicd [114].

### 7.2. Activation during Endocytic and Lysosomal Trafficking of the Notch Receptor

Besides mutations in the Notch receptor that induce the spontaneous unfolding of the ECD of Notch, alternative shedding mechanisms have been proposed. Upon endosomal trafficking the Notch receptor can be transported towards the lysosomes for degradation. Therefore, another possible mechanism of ECD shedding could be the degradation of the ECD by hydrolases, to which also Adam metalloproteases belong, and subsequent γ-secretase cleavage, which are both present in lysosomes, resulting in the active form of Nicd, which may be released and translocated towards the nucleus [50]. Notably, during the incorporation of the Notch receptor into endosomes, the Notch receptor is located to the limiting membrane on the late endosome, resulting in the intracellular domain of Notch protruding into the cytosol [48,49,50]. During this intracellular trafficking, γ-secretase may cleave the receptor at the free S3-site, releasing the active Nicd into the cytosol, which can subsequently travel towards the nucleus and activate its downstream targets. However, excessive shortening of the extracellular domain of Notch may be still required for γ-secretase cleavage, since γ-secretase cleavage increases upon a shorter Notch receptor substrate [99].

Furthermore, defects in genes involved in the ubiquitination and lysosomal degradation of the Notch receptor may in a ligand-independent manner, activate Notch signaling. For example, loss of Numb decreases Notch localization towards late endosomes and leads to an accumulation of Notch at the cell surface, due to increased recycling of the Notch receptor, which results in reduced degradation of the Notch receptor [38]. Interestingly, blocking the fusion between late endosomes and lysosomes or by reducing their acidification by Chloroquine, Bafilomycin A1, or NH_4_Cl results in an accumulation of Notch in late endosomes and lysosomes, down-regulating ligand-independent Notch signaling [75,76,77,78,83].

These data suggest that endosomal fusion is required for ligand-independent Notch signaling. Moreover, additional fusions of intracellular vesicles may be affected by these treatments leading to different blockades in endocytic trafficking. Together these data show that intracellular trafficking is important for ligand-independent Notch activation and that defects at different steps of trafficking can either support or inhibit ligand-independent signal transduction.

### 7.3. Changes in the Endosomal-Lysosomal Environment

**Figure 5:**
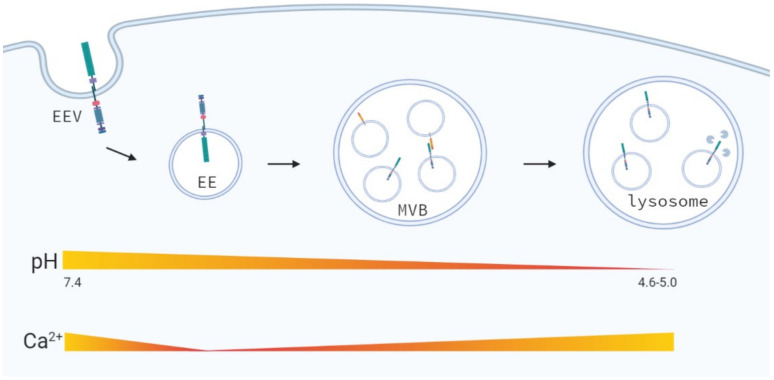
Changes in the ion-concentrations during intracellular trafficking. The vesicular pH decreases upon endocytic trafficking due to import of H+-ions leading to gradient acidification. Ca^2+^-ion concentrations initially decrease during early endosomal trafficking and gradually increase during late endosomal trafficking reaching the highest Ca^2+^-ion concentration in the lysosomes. Adjusted from Scott and Gruenberg [115].

During intracellular trafficking changes in different ion-concentrations occur in the endosomal-lysosomal microenvironment (Figure 5) [115]. During endocytic trafficking the pH of endosomal compartments becomes gradually more acidic [116], due to increased H^+^-ion transport towards these vesicles by V-ATPase activity [77]. Changes in pH affect protein–protein interactions, enzymatic activity and protein degradation and may affect the activity of the enzymes regulating the proteolytic cleavage of the Notch receptor. Therefore, the acidic environment in endosomes and lysosomes may promote the dissociation the ECD of the Notch receptor and result in ligand-independent activation of Notch. In addition to the gradual increase in acidity of intracellular vesicles, Ca^2+^-ion concentrations change during endocytic transport. Calcium ions are high at the plasma membrane and rapidly decrease upon internalization and formation of early endosomes, from which a calcium gradient builds-up again towards lysosomal trafficking [117,118]. High calcium in the lysosomes is required to induce a calcium efflux to ensure fusion of lysosomes with late endosomes and autophagosomes [119]. Calcium depletion in early endosomes may, however, decrease the stability of the Notch receptor, especially since the LNR repeats in the NRR require Ca^2+^ to stay intact [120]. This is in line with the fact that Ca^2+^ chelators (i.e., EDTA) are potent activators of Notch signaling [121,122] resulting in unmasking of the Notch1 S2-cleavage site, leading to Notch activation [3].

### 7.4. Alternative S2 Cleavage?

As mentioned before, ligand-independent Notch signaling does not require Adam10 for its activation, however, can be cleaved by Adam10 at Val1711. Van Tetering et al. showed that both genetic and pharmacologic inhibition of metalloprotease activity blocks ligand-independent Notch receptor cleavage at Val1711, however, does not block general S2-cleavage and activation of the Notch receptor [3]. While the majority of Val1711-cleaved Notch receptors occurred at the plasma membrane, a fraction of Val1711-cleaved Notch receptors also localized to cytoplasmic vesicles. However, the identity of these intracellular vesicles has not yet been revealed and the exact site(s) of alternative S2-cleavage of the Notch receptor and the protease(s) involved in the absence of Adam10 have not been elucidated. Taking a similar approach, Hounjet et al. identified that Val1744-cleaved Nicd can accumulate, when cells are treated with chloroquine, in LC3B+ auto-phagosomal vesicles [83]. Notably a large fraction of Val1744+ vesicles did not co-localize with LC3B-GFP demonstrating other unidentified intracellular vesicles, which contain S3-cleaved Nicd.

The hypothesis that S2-cleavage occurs at the plasma membrane is supported by data showing that: (1) S2-cleaved Notch remains at the plasma membrane in γ-secretase mutant fly cells [123], (2) S2-cleaved Notch, which harbors a mutated ubiquitination site required for internalization of the receptor, can still be cleaved by γ-secretase into active Nicd [123], and (3) Adam10 and Adam17 are predominantly expressed at the cell surface [124]. Others show that Adam10 is expressed at the cell membrane, but that the majority of the pro-enzyme is expressed in the Golgi [125]. In addition, Dornier et al. showed that tetraspanins regulate Adam10 trafficking from the ER to the cell surface and that tetraspanins promote ER exit and surface expression of Adam10 thereby stimulating Notch activation [126,127]. In contrast, there are also reports showing that S2-cleavage can occur in intracellular vesicles [57,128]. Chastagner et al. showed that ligand-bound Notch is internalized by Dtx4 ubiquitination, which results in Rab5-expressing early endosomes containing Notch1 and Adam10 in which Notch is first internalized before Adam10 processing [57]. In addition, S2-cleavage may even be circumvented during ligand-independent Notch signaling, as γ-secretase cleavage does not have strong sequence requirements in the transmembrane domain of Notch. Moreover, alternative ECD shedding may result in a short enough ECD of Notch1 (<300 aa) to be recognized by γ-secretase, which activity increases when the substrate is decreased in size [99]. Whether non-S2-cleaved Notch can be a γ-secretase substrate remains unknown.

### 7.5. Alternative S3 Cleavage?

Currently, the role of intracellular trafficking of Notch in γ-secretase cleavage is understudied. First, a mutation in the mono-ubiquitination site at Lys1749 of a Next-like fragment results in a loss of γ-secretase cleavage and the accumulation of Notch in the endosomes [28]. These data suggest a requirement of endocytic trafficking following S2-cleavage of Notch. Furthermore, endocytosis was required prior to γ-secretase cleavage since inhibition of endocytosis prevented S3-cleavage.

The γ-secretase complex is expressed at the plasma membrane but also on endosomes, lysosomes, ER, and Golgi [129,130]. Gamma-secretase is a membrane protein complex comprised of presenilin, nicastrin, Aph-1, and Pen-2. Mammalian cells encode for two presenilins, PSEN1 and PSEN2, the catalytic subunit of the γ-secretase complex. PSEN1 or PSEN2 containing γ-secretases, show distinct subcellular localization and activity. PSEN1-expressing γ-secretase complexes predominantly localize to the plasma membrane, while PSEN2-expressing γ-secretase complexes are enriched to the late endosomes and lysosomes [23]. This subcellular localization of presenilins is regulated by an Acidic-Dileucine sorting motif. Disruption of this motif results in a translocation of PSEN2-containing γ-secretase activity to the plasma membrane.

In addition to the intracellular localization of the γ-secretase complex, its activity has also been reported to be affected by pH. During endocytic trafficking cargo proteins experience a reducing pH gradient towards the lysosomes. Indeed, γ-secretase activity was shown to be more efficient in the acidic environment of endosomes and lysosomes compared to the plasma membrane [130]. Furthermore, the differences in pH also affect γ-secretase cleavage sites in the Notch transmembrane domain [131]. Tagami and colleagues showed for the first time that γ-secretase is able to cleave the Notch receptor at two different amino acid sites in mammalian cells for both ligand-dependent and independent Notch signaling [132]. These two different cleavages by γ-secretase result in the release of the Nicd-V (Gly1743-Val1744) or the novel Nicd-S (Leu1746-Ser1747), from which the latter is more produced (especially when the internalization rate is high), less stable, and predominantly expressed in endosomes, however, Nicd-V was shown to be a stronger activator of the Hes1 promoter. Blockage of the acidification of endosomes and lysosomes by Bafilomycin A1 treatment results in a shift in Nicd cleavage in which the Nicd-V becomes the major site of cleavage. Thus, vesicular pH and trafficking can have a major impact on the fate of Notch molecules in vitro.

Besides endopeptidase activity leading to alternative S3-cleavages, γ-secretase also possesses carboxy-peptidase activity [133]. Okochi and colleagues showed that the Notch transmembrane is cleaved twice; first at the S3-cleavage site which depends on endopeptidase activity (S3ε) and secondly at the S4-cleavage site (Ala1731-Ala1732) which depends on the carboxy-peptidase activity of γ-secretase (S4γ) [134,135]. Importantly, γ-secretase cleavage at the cytoplasmic leaflet (S3) is required prior to S4-cleavage [136]. This dual intra-membrane cleavage by γ-secretase results in the release of a S4-Notch peptide, which is secreted and does not accumulate intracellularly. The significance of this peptide has not been investigated but perhaps acts as a mechanism to clear processed remnants of the Notch receptor from the cell.

Together these data show that there are multiple possible mechanisms for ligand-independent Notch signaling and that intracellular trafficking plays an important role in all proposed mechanisms.

## 8. Therapeutic Targeting of Ligand-Independent Notch Signaling

Ectopic activation of Notch signaling is a driver in many human malignancies, including T-ALL, breast cancer, and lung cancer; hence, Notch inhibition is a high priority clinical target. However, inhibition of the Notch pathway as a therapeutic approach faces challenges due to its essential function in the maintenance of adult tissues. The lack of predictive biomarkers for patient selection, the fact that Val1744 is not a good marker for Notch activity as Val1744 levels do not necessarily correlate with Notch1 activity [83], the sparse availability of robust pharmacological markers to monitor on-target activity [137], and the pleiotropic effect of current pan-Notch inhibitors targeting multiple Notch family members (both with GSI, as well as, ligand neutralization) lead to low anti-tumor activity and dose-limiting toxicities in normal tissues.

However, toxicity due to γ-secretase inhibition depends on the schedule of the treatment. Intermittent scheduling results in strong modulation of a Notch gene signature, including down-regulation of *Notch1* and its target genes, evidence of anti-tumor activity and low toxicity in phase I clinical trials [137,138]. Moreover, the recent availability of a Notch1 receptor specific monoclonal antibody showed promising results in preclinical studies, including inhibition of tumor growth, deregulation of angiogenesis, and reduced intestinal toxicity compared to pan-Notch inhibition in preclinical models [139]. Other monoclonal antibodies that specifically recognize activated Notch receptors have also shown promising results in preclinical studies [140,141], however, failed in clinical trials due to intolerable toxicities and lacking of anti-tumor efficacy. Tarextumab, a monoclonal antibody targeting Notch2 and Notch3, was well tolerated using intermittent scheduling and showed inhibition of Notch signaling in a phase I trial [142]. Unfortunately, the addition of Tarextumab to chemotherapy as a therapeutic strategy in pancreatic adenocarcinoma failed to improve patient outcome [143]. Thus, despite the overwhelming preclinical efficacy to date no clinical product exists that can safely and effectively target Notch in patients. Therefore, it is essential to obtain a better understanding in the mechanisms behind this ectopic, ligand-independent activation of Notch signaling and the role of intracellular trafficking, especially in the differences between ligand-dependent and independent signaling. If such differences could be exploited this would allow targeting of oncogenic ligand-independent Notch signaling without affecting physiologic Notch signaling in adult tissues.

Recently, Hounjet et al. published a repurposed drug use approach in which T-ALL cell lines were treated with chloroquine in combination with γ-secretase inhibition [83]. Chloroquine sensitized oncogenic Notch1 driven human T-ALL to GSI resulting in decreased T-ALL cell viability and proliferation. In these Notch1 driven T-ALL cells chloroquine interfered with the intracellular trafficking and processing of ligand-independent Notch1 receptors, leading to the accumulation of full length but also S2- and S3-cleaved Notch1 and down-regulation Notch target gene expression. Importantly, this reduction in Notch1 target gene expression was not observed in GSI-resistant T-ALL cells with wild-type Notch signaling. Moreover, while GSI treatment led to an accumulation of oncogenic Notch1 at the plasma membrane, when combined with chloroquine, Notch1 accumulated in LC3B+ autophagosomes but also in other intracellular vesicles, yet to be identified. In addition, Notch receptor expression at the plasma membrane was decreased upon chloroquine treatment, indicating a defect in receptor internalization or recycling. The efficacy of chloroquine in the treatment of T-ALL (in combination with GSI) in preclinical studies remains to be studied. Importantly, the synergistic interaction between chloroquine and GSI argues that strongly reduced concentrations of the individual drugs retain the therapeutic efficacy but with less toxicity providing a therapeutic window for safe Notch targeting. Mechanistically the combined treatment of GSI and chloroquine led to a strong increase in cytoplasmic ROS. Whether this increase in ROS caused the increased cell death is under current investigation.

Maes and colleagues showed that chloroquine can also increase endogenous Notch1 signaling in endothelial cells, inducing tumor vessel normalization in tumors [144]. They report that Notch1 receptors accumulate in late endosomal vesicles upon chloroquine treatment and show a slow and sustained increase in Next and Nicd levels, which may be due to reduced lysosomal degradation, while ligand-induced activation leads to an acute and transient increase in Nicd levels. However, Kobia et al. showed that V-ATPase inhibition reduces ligand-dependent Notch signaling in human breast cells [76]. Although Hounjet et al. show that inhibition of the lysosomal fusion of endosomes, by changing the intracellular pH by chloroquine, inhibits ligand-independent Notch signaling, it remains controversial whether ligand-dependent Notch signaling is also effected by chloroquine treatment and may depend on the cellular background and might be tissue specific.

## 9. Concluding Remarks

We conclude that intracellular trafficking is an important regulator of Notch activation. In *Drosophila* inhibition of early endosomal trafficking and endosomal-lysosomal fusion inhibits Notch signaling activation, while accumulation of Notch in maturing endosomes results in Notch signaling activation. In mammals, intracellular trafficking seems to play a similar role in the regulation of Notch signaling, although the expression of several orthologs of endosomal regulation may induce redundancy and makes studying the effects of loss-of-function more complex. Importantly, deregulation of the pH in intracellular vesicles in mammals results in an accumulation of (cleaved) Notch receptors in late endosomes and lysosomes, leading to decreased ligand-independent signaling. In addition, the release of Notch ligands, receptors, and regulators in extracellular vesicles and internalization and Notch activation in recipient cells in mammals adds another layer of complexity to the role and importance of intracellular trafficking in the activation of mammalian Notch signaling.

While mutations, rearrangements, and fusions of *Notch* leading to Notch activation are well defined, the Notch activation mechanisms in other malignancies remains unclear. The recruitment of Adam10 and γ-secretase complexes towards intracellular vesicles strengthens the model of intracellular Notch receptor processing and activation. Intracellular pH and ion-concentrations (i.e., Ca^2+^_,_ Zn^2+^) may play a critical role in the regulation of Notch activation either by directly affecting the stability of the receptor itself or indirectly by affecting the activity and/or cleavage site precision of proteolytic enzymes required for ligand-independent Notch activation.

Overall, the exact differences between ligand-dependent and independent Notch signaling in mammals and how these are regulated remain inconclusive. Undoubtedly, additional regulators of Notch cleavage and activation exist that control or drive these distinct modes of activation. If intracellular trafficking indeed differentially regulates ligand-independent Notch signaling, endosomal targeting could be exploited to achieve specific inhibition of ligand-independent Notch signaling, without affecting the ligand-dependent signal. In addition, it will be interesting to see under what developmental programs Notch is activated in the absence of ligand in mammalian cells and how this mechanism prevents inappropriate Notch signaling during development and in adult tissues. Such regulators are prime candidates for curtailing aberrant Notch signaling in cancer, guiding us towards a more precise approach, keeping an open eye to the future for targeting of Notch signaling in cancer.

## Figures and Tables

**Table 1:** Key regulators of intracellular trafficking and their effects on Notch signaling upon loss-of-function in both *Drosophila* and mammals.

	*Protein Drosophila/* *Mammalian*	*Function*	*Loss of Function* *Drosophila*	*Loss of Function* *Mammals*
*Internalization*	Shibire/Dynamin	Endosomal vesicle formation	↓Notch signaling ↑Notch receptor expression at the plasma membrane	Dynamin 1 =↓NotchΔE internalization, no effect on Val1744 levels in the nucleus (HeLa cells), γ-secretase-mediated Next cleavage Dynamin 2 =↓γ-secretase cleavage of Notch1-ΔE-TM
	Clathrin heavy chain	Endosomal vesicle formation	↓Ligand-dependent Notch signaling	↓NotchΔE internalization, no effect on Val1744 levels in the nucleus
	Crumbs/Crb1	Prevents Notch receptor internalization and activation	↑Internalization of Notch ↑Ectopic ligand-independent Notch signaling	
*Recycling*	Rme-8/ Dnajc13	Endosomal sorting and recycling	↑Notch in enlarged Rab4+ endosomes ↓Notch signaling	
	Numb	Stimulates Notch receptor trafficking towards MVBs (degradation) instead of recycling endosomes	↓Notch in late endosomes ↑Notch at the plasma membrane ↓Ubiquitination of Nicd ↑Notch signaling	↑Notch signaling ~50% of all human breast cancers ~30% of all human NSCLC
	Lethal giant larvae/ Lgl1-4	Inhibits Notch signaling by controlling the asymmetric localization of Numb	↑Notch signaling ↑Nicd	↑Notch signaling ↑Nicd
*Early endosomes*	Rab5, Avl/Syntaxin 7	Entry into early endosomes	↓Notch signaling ↑Notch receptor expression at the plasma membrane	
	Deltex (dx)	E3-ligase, mono-ubiquitination of Notch-icd Endosomal stabilization and activation of Notch	↓Notch signaling ↓Notch internalization	↑Notch signaling DTX1^−/−^2^−/−^ mutant mice = no defects in Notch dependent T-cell development
	Suppressor of Deltex (Su (dx))/Itch	E3-ligase, poly-ubiquitination and lysosomal degradation of Notch	↑Notch signaling	Itch mutant mice = severe autoimmune disease Itch deficiency in humans = severe autoimmune disease and morphologic and developmental abnormalities
*Late endosomes*	ESCRT	Maturation of early endosomes into MVBs	↑Notch in endosomes ↑Ligand-independent Notch signaling	CHMP5 (ESCRT-III)—No activation of Notch signaling Vps25 (ESCRT-II)—No activation of Notch signaling Tsg101 (ESCRT-I)—No activation of Notch signaling (in vitro), early embryonic lethal (in vivo) BBS1/3/4—↑ligand-independent Notch signaling, accumulation of Notch in late endosomes
	Lgd/Cc2d1a	Maturation of early endosomes into MVBs	↑Notch in early endosomes ↑Ligand-independent Notch signaling	↑Endosomal size in intestinal Cc2d1a mutant mice, ~ Notch signaling Loss of both orthologs is not validated yet
	Hrs/Hgs	Component ESCRT-0, recognizes ubiquitinated proteins and facilitates transport from early to late endosome	↑Notch in early endosomes ↓Notch signaling	
*Nuclear translocation*	Importin α Importin β1	Nuclear import of Nicd	↓Nuclear localization of Notch ↑Notch in the cytoplasm	↓Nuclear localization of Notch

## Data Availability

Not applicable.
